# Dynamics of Ligand Binding Sites and Chloride Penetration in a Bitter Taste GPCR

**DOI:** 10.1021/acs.jcim.6c00140

**Published:** 2026-09-03

**Authors:** Alon Rainish, Liel Sapir, Masha Y. Niv

**Affiliations:** † The Institute of Biochemistry, Food Science and Nutrition, The Robert H. Smith Faculty of Agriculture, Food and Environment, 229 Herzl Street, Rehovot 76100001, Israel; ‡ The Fritz Haber Center for Molecular Dynamics, The Hebrew University, Jerusalem 919041, Israel; § Department of Chemistry and the Institute of Nanotechnology and Advanced Materials, 26731 Bar-Ilan University, Max and Anna Webb, Ramat Gan 5290002, Israel

## Abstract

Taste perception strongly influences food choice, drug compliance, and dietary behavior. Bitter taste receptors belong to a subfamily of Family A GPCRs, while having unique features and deviations from conserved motifs. Recent cryo-EM structures revealed that the human bitter taste receptor TAS2R14 contains not only the canonical extracellular binding site, but also a novel intracellular site, raising questions about their interplay. Here, we performed all-atom molecular dynamics simulations of TAS2R14 in an explicit membrane to probe different receptor states: both cholesterol (extracellular) and aristolochic acid (intracellular) bound, only cholesterol bound, only aristolochic acid bound, and the apo receptor, with and without the G protein. Ligand occupancy at either site reshaped the volume, hydration, and conformational plasticity of both pockets and modulated the relative proximity of transmembrane helices 3 and 6. In simulations lacking the intracellular agonist but containing cholesterol, we observed intracellular entry of chloride ions and their long-lived interaction with the TAS2R-conserved R55 (BW position 2.50), revealing a charge-inverted interaction relative to the D2.50-sodium interaction described in many class A GPCRs. Apo simulations without G protein showed shorter-lived chloride visits to the same region. Together, these findings point to the intracellular pocket as a promising target for future mechanistic and ligand-design studies on bitter taste GPCRs.

## Graphical abstract

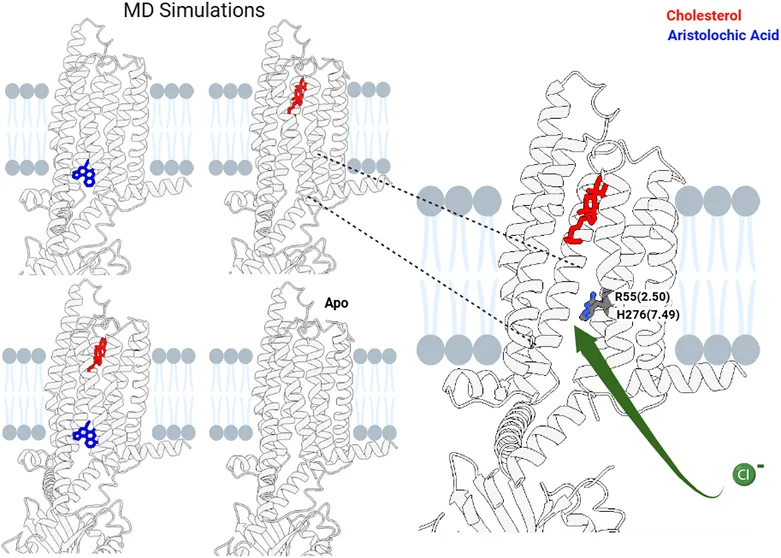

## Introduction

Bitter taste receptors (TAS2Rs or T2Rs) are a subfamily of G protein-coupled receptors (GPCRs) responsible for mediating the perception of bitterness.^1^ In humans, 25 functional TAS2Rs have been identified, with the 26th member of this subfamily, TAS2R2, reported as functional in some populations.^2,3^ TAS2R14 is the most broadly tuned bitter taste receptor, activated by a wide range of chemically diverse ligands.^4^ TAS2R14 is expressed not only in the oral cavity but also in various extra-oral tissues, including the heart, lungs, reproductive system and more.^5^ Its widespread expression suggests additional physiological roles beyond taste perception. TAS2R14 has been implicated in several disease contexts, including associations with colorectal^6^ and pancreatic^7^ cancers, positioning it as a potential therapeutic target.

The recent cryo-electron microscopy (cryo-EM) structures of TAS2R14 have revealed unexpected features, most notably an additional, intracellular, ligand-binding site.^8–11^ This ligand binding was neither predicted by computational models^12,13^ nor observed in other TAS2R structures that have been solved so far, namely TAS2R46,^14^ TAS2R16,^15^ TAS2R43 ^16^ and TAS2R4.^17^ The presence of this unusual pocket challenges traditional views of GPCR agonist binding and activation, raising questions about its functional significance and the potential interplay between intra and extracellular environments^18^. Advances in molecular dynamics (MD) have demonstrated that unbiased, multimicrosecond simulations of GPCRs can reliably sample key conformational substates and reveal ion or lipid allosteric sites (e.g., GPCRmd, which captures the dynamics of the three-dimensional (3D) GPCR-ome).^19,20^ While some structures show the extracellular site being occupied by the same intracellular ligand (PDB ID: 8RQL,^8^ 9IIX, 9IIW^11^) or unoccupied (8XQR^9^), cholesterol has been observed at the extracellular binding site of several TAS2R14 structures.^9–11^ Basal receptor activity was shown to depend on cholesterol occupancy of the extracellular pocket, which can be reduced upon cholesterol depletion and restored following cholesterol replenishment.^10^ To explore the behavior of this novel intracellular pocket, we applied molecular dynamics simulations to the recently solved cryo-EM structure of TAS2R14 bound to aristolochic acid (PDB: 8XQO),^9^ in which aristolochic acid (AA) occupies the intracellular site, while cholesterol (CLR) is bound to the extracellular site. Our study used MD simulations to investigate the stability of the intracellular pocket and its conformations depending on the extracellular binding site. Simulations were performed for different states, namely: with both sites occupied, a single site occupied, and the apo state (with and without the Gα protein). The analysis showed that ligand at one site may affect the volume of the other site. Unexpectedly, we found that the unoccupied intracellular site allows for intracellular entry of a chloride ion, leading to its interaction with the TAS2R-conserved arginine at the 2.50 Ballesteros-Weinstein (BW) position.^21^

## Materials and Methods

### Preparation and Simulation

We initially prepared four systems for the TAS2R14 MD simulations: a system of the experimental structure PDB ID: 8XQO with both ligands (“Both ligands”), one with only cholesterol in the extracellular site (“only CLR”), one with only aristolochic acid in the intracellular site (“only AA”) and one apo state (“Apo”). These systems include the Gα subunit. An additional apo system was simulated without the Gα subunit.

All MD systems were prepared using the CHARMM-GUI Membrane Builder,^22^ using the histidine protonation state predicted by MolProbity^23^ (version 3.7.201124) (see Supporting Information). Missing loops ECL2 (residues 162–172) and ICL3 (residues 217–226) were added based on the AlphaFold model (AF-Q9NYV8-F1-v6).^24^ Protein preparation was performed using the Protein Preparation Wizard (Schrödinger Release 2025–1, Schrödinger, LLC, New York, NY, 2025) within the Schrödinger Suite. The complexes were oriented in the membrane using the default OPM alignment, and embedded in a palmitoyl-oleoyl-phosphatidylcholine (POPC) lipid bilayer, with a rectangular box extending at least 15 Å from the protein in each direction along the membrane.

All systems were solvated with CHARMM TIP3P water and supplemented with 50 mM NaCl. While this concentration is lower than typical extracellular NaCl levels, it falls within the range^25^ reported for intracellular chloride in some taste cells^26^ and olfactory sensory cells^27^ and provides a stable ionic environment for examining ion-receptor interactions.^28^ This resulted in approximately 60,300 water molecules and 128 Na^+^/Cl^–^ ions per system, depending on system size. Energy minimization was performed using a multistep steepest descent protocol with gradually released positional and dihedral restraints, following the standard CHARMM-GUI workflow (Table S1).

Systems were subsequently equilibrated under NVT and NPT conditions with progressively decreasing positional restraints (Table S2). Temperature was maintained at 310 K using the velocity-rescale thermostat,^29^ and pressure was controlled semi-isotropically at 1 bar using the Berendsen barostat^30^ during early equilibration and the Parrinello–Rahman barostat^31^ in later stages. Production simulations were carried out under NPT conditions using a 2 fs time step, the velocity-rescale thermostat, and the Parrinello–Rahman barostat. Long-range electrostatics were treated using the particle mesh Ewald method, and all bonds involving hydrogen atoms were constrained using LINCS. Each system was simulated for 2 μs per replica with three independent replicates (Table S3). Ligand parameters and topologies were generated using the Ligand Reader & Modeler module of the CHARMM-GUI^22^ web server. Force field parameters were derived from the CHARMM General Force Field (CGenFF)^32^ ensuring compatibility with the CHARMM36m force field used for the protein. All-atom molecular dynamics simulations were carried out using GROMACS version 2021.5.^33^ The system was parametrized using the CHARMM36m force field. The structural integrity of the ligand force field parameters was validated through structural alignment and RMSD analysis against reference coordinates (Figure S1).

### Volume Calculation

Cavity volume calculations were performed using the POVME 3.0 software package^34^ with a grid spacing of 1.0 Å and a distance cutoff of 1.09 Å. For the intracellular (AA) binding site, the inclusion region consisted of a 10 × 10 × 12 Å box combined with a 6.0 Å inclusion sphere, centered at (141.10, 144.45, 145.60). For the extracellular (CLR) site, a 16 × 16 × 16 Å box was used with a 4.0 Å inclusion sphere, centered at (129.03, 127.39, 164.88). To ensure reproducibility and consistent sampling across frames, inclusion regions for each pocket were defined based on the initial ligand coordinates. No additional exclusion masks were applied beyond standard automatic cavity filtering.

### Root Mean Square Deviation (RMSD) Analysis

RMSD analysis was used to assess the structural stability of TAS2R14 during MD simulations. Calculations were performed with Visual Molecular Dynamics (VMD) v1.9.4 ^35^ using the MDanalysis python package, incorporating periodic boundary condition correction, recentering, and alignment to the reference cryo-EM structure PDB ID: 8XQO. Receptor protein backbone atoms were used for alignment and RMSD calculation. Ligand RMSD was computed based on heavy atoms after aligning the receptor backbone in each frame to the PDB structure, in order to assess relative movement within the binding site (Figure S2). While a single ligand dissociation event was captured in one of the AA-bound trajectories, the overall stability of the ligand coordinates and binding footprints across all other replicas indicates this was a rare stochastic event rather than a systemic parametrization artifact.

### Contact Analysis

Protein–ligand interactions were quantified using the MDAnalysis^36^ library by calculating the contact counts and frequencies between the ligand and receptor residues. A contact was defined as any residue having at least one atom within a 3.5 Å distance cutoff of any ligand atom. Contact frequencies were calculated as the percentage of simulation frames in which a contact was maintained. Analysis was performed across all three independent replicates for each system, and results were averaged to determine the mean occupancy and associated standard deviations. For comparative visualization, residues were filtered to include only those exhibiting a contact frequency of ≥ 50% in at least one of the evaluated states, which were then plotted alongside their corresponding binding energy decomposition profiles (Figure S3).

### MM/GBSA Binding Energy Calculations

Binding free energies and residue-specific decompositions were calculated using gmx_MMPBSA,^37^ version v1.6.3. Analysis was performed on the production trajectories with an interval of 10 frames. The polar solvation contribution was estimated using the Generalized Born model (igb = 2) with a salt concentration of 50 mM. Per-residue energy decomposition (idecomp = 1) was employed to quantify individual amino acid contributions to the binding affinity. All calculations utilized the same force field parameters as the MD simulations. Results are shown in Figure S3.

### Root Mean Square Fluctuation (RMSF) Analysis

RMSF analysis was performed by MDAnalysis^36^ (v2.0+) to quantify residue-level flexibility in TAS2R14. The simulation trajectory, corrected for periodic boundary conditions and aligned to the input structure, was analyzed for Cα atoms of the receptor. RMSF values were calculated separately for each replica (Figure S4) with the resulting mean values reported in the main text.

### Water Molecule Count Analysis

Water molecules within 3 Å of the ligand were counted throughout the simulation using a custom Tcl script in VMD v1.9.4. For all systems, frames were first aligned to the initial cryo-EM receptor backbone (Cα atoms). In ligand-bound systems, the selection volume dynamically tracked the ligand heavy atoms in each frame. In ligand-free states the counting region was anchored to the coordinates of the initial cryo-EM ligand-binding pose. This setup ensured that the reference frame tracked the local pocket movement rather than remaining static in Cartesian space. The same alignment parameters and selection criteria were applied to generate 3D water density maps using the VMD VolMap^38^ plugin.

### PCA Analysis

Conformational dynamics of TAS2R14 were analyzed by principal component analysis (PCA) on Cα atoms, using MDAnalysis for trajectory handling and coordinate extraction, and scikit-learn^39^ for computing the PCA and projecting the structures onto principal component space. Because ECL2 and parts of ICL3 were unresolved in the starting structure and subsequently modeled computationally, their high intrinsic flexibility introduces significant conformational noise. Consequently, both loops were omitted from both the trajectory alignment and the subsequent principal component analysis. Trajectories were first aligned to the prepared PDB to remove overall rotation and translation, and frames were sampled every 0.2 ns. PCA was performed on concatenated coordinates from all replicates and all four systems (with the ECL2 and ICL3 removed). Density distributions along the first two principal components were computed using Gaussian Kernel Density Estimation to map the free energy landscape, and representative frames were extracted from distinct, system-specific conformational clusters for detailed structural inspection.

### Distance and Chloride Occupancy Analysis

Distances between the Cα atoms of residues V230 (BW number 6.35) and Y107 (BW number 3.50) were measured along the trajectories using a custom Tcl script in VMD, sampling every 0.2 ns to reduce data redundancy. Chloride occupancy near the R55 (2.50) position was similarly quantified by identifying all chloride ions within 4 Å of R55 in each frame, and the fraction of frames in which an ion occupied that position was calculated. All analyses were performed across replicate trajectories for each simulation condition (individual results for all replicates are provided in Table S4 and Figure S9).

### Electrostatic Surface Potential Calculations

Electrostatic surface potential calculations were performed to assess the electrostatic environment of the intracellular region of TAS2R14 prior to chloride entry. A representative snapshot was extracted from the MD trajectory before the appearance of chloride ions in the intracellular pocket. To prepare the structure for continuum electrostatic calculations, the snapshot was processed using PDB2PQR,^40^ employing the CHARMM force field parameters at pH 7.0. Consistent with the initial system setup described above, histidine protonation states were manually set to match the MolProbity^23^-determined tautomeric assignments, as detailed in Supporting Information. Electrostatic potentials were calculated by solving the linearized Poisson–Boltzmann equation using APBS,^41^ version 3.4.1. The resulting electrostatic potential maps (dx files) were visualized in UCSF ChimeraX^42^ (version 1.4) and mapped onto the solvent-excluded (molecular) surface of the receptor. Surface coloring was applied over a range of −10 to +10 kT/e to highlight regions of positive and negative electrostatic potential.

## Results and Discussion

To investigate the function of the intracellular binding site of TAS2R14 and its relationship with the extracellular site, we performed MD simulations using the recently solved structure of TAS2R14 (PDB: 8XQO), which includes aristolochic acid (AA) bound to the intracellular site and cholesterol (CLR) bound to the extracellular site. Four distinct receptor states were simulated, each in triplicate for 2 μs: Both ligands bound (AA and CLR), only AA (CLR removed), only CLR (AA removed) and Apo (with Gα, both ligands removed) (Figure 1, Table S3). Unless otherwise specified, all analyses were performed on three replicates of each tested binding state, and results are presented as the average of the three. We analyzed differences between the states focusing on both binding sites using structural and dynamic measures including volume, conformational changes, ion behavior, water distribution, and movement of ligands and protein.

**Figure 1:**
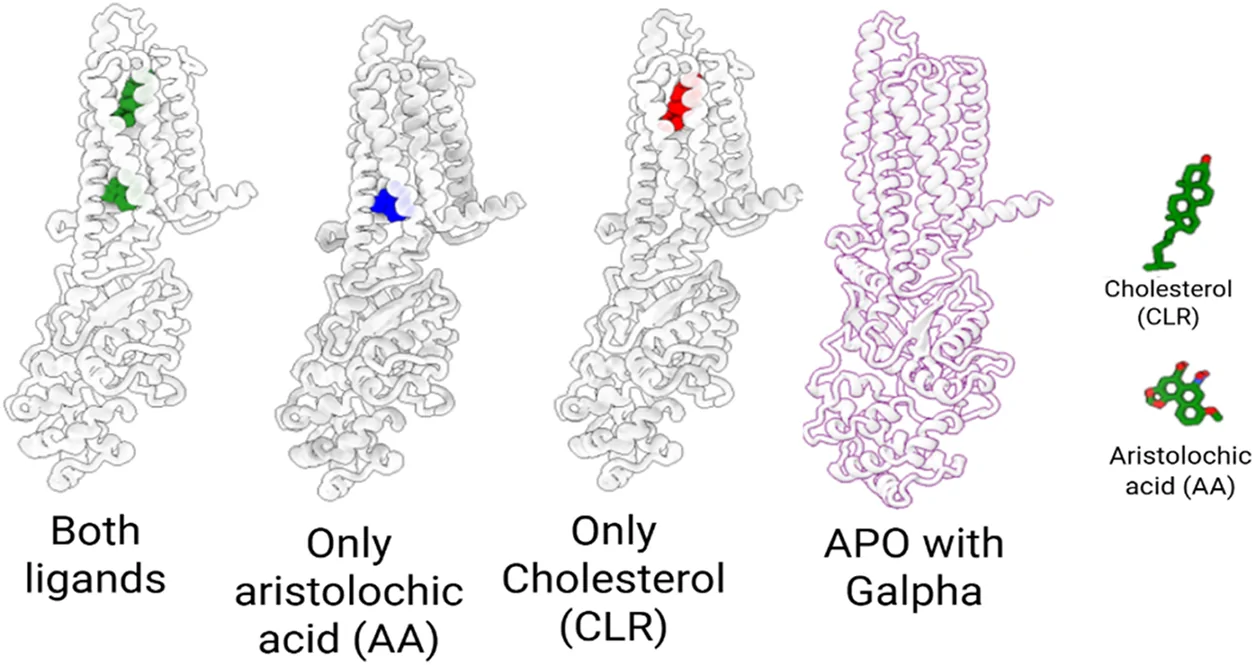
Simulated systems and the ligands bound to each of the sites. The color scheme to distinguish between the different systems is used throughout.

### Pocket Volumes and Ligand Effects

The volumes of the intracellular and extracellular binding pockets are depicted in Figure 2. States containing a bound ligand in the extracellular site exhibited larger volumes with the only CLR state showing the greatest expansion. Cholesterol-free states displayed lower volumes in the extracellular site characterized by broader distributions. In the intracellular site, states with a bound ligand had larger volumes, with the dual-ligand state having the largest intracellular volume, and where the higher volume states are characterized by a broader distribution.

**Figure 2:**
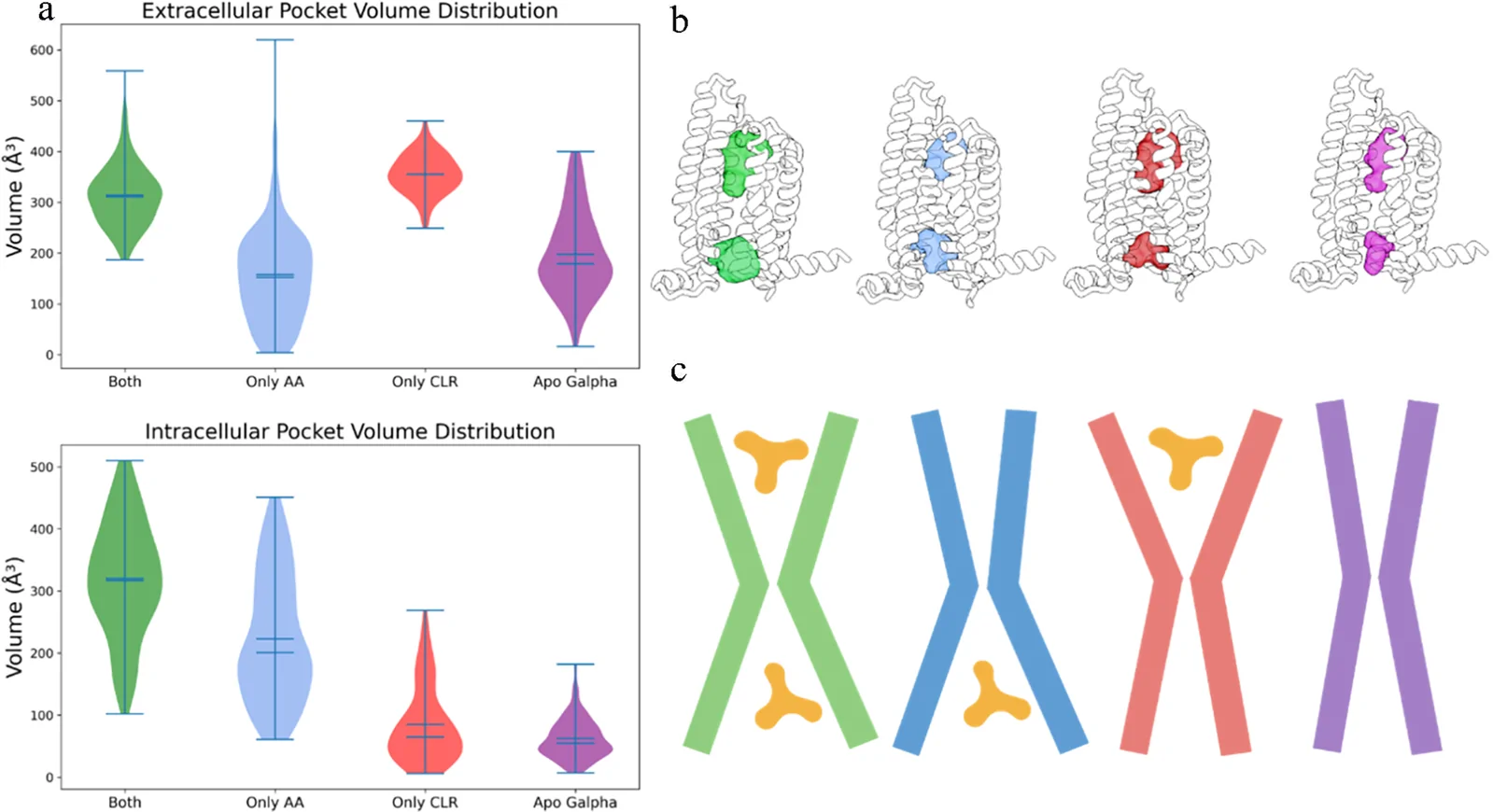
Volume analysis of the binding sites. (a) Violin plots showing the pocket volume distributions for the extracellular (top) and intracellular (bottom) binding sites across different receptor states. (b) Structural representations of representative snapshots of the measured pocket volumes in each state, using the same color scheme: both ligands bound (green), only aristolochic acid bound at the intracellular site (blue), only cholesterol bound at the extracellular site (red), and apo state with the Gα subunit present (purple). (c) Schematic representation of the binding sites and ligand positions.

The distributions highlight differences between the two binding pockets. In the extracellular site, CLR-bound states show larger but tighter distributions whereas CLR-free states are smaller but more variable, consistent with greater flexibility. In the intracellular site, AA-bound states display higher volumes with broader distributions, indicating a more open and dynamic conformation compared to the more compact AA-free states. These trends point to ligand-dependent effects on pocket flexibility. Panel (b) maps the measured volume differences onto the receptor structure, visually showing which regions of the extracellular and intracellular pockets expand in each ligand state. Panel (c) provides a simplified schematic that captures these shifts, emphasizing the overall trend of pocket opening or narrowing depending on ligand binding. The differences between the sites suggest the extracellular site exhibits a broader volume distribution when unoccupied, compared to the intracellular site.

Beyond these volumetric shifts, we observed that in the presence of cholesterol, some of the intracellular site interactions with AA are stronger compared to the cholesterol-free condition, as detailed in the subsequent residue-specific energy and contact analysis (Figure S3). We next explored the structural details underlying the volume changes.

### Ligand-Induced Conformational Changes in TAS2R14

PCA analysis was performed on the receptor’s Cα atoms using the concatenated trajectories from all systems and replicates in order to evaluate allosteric changes between the states. The analysis reveals that the different systems occupy distinct conformational regions in PCA space. Notably, the Apo and dual-bound states exhibit the least conformational diversity, as reflected by their more compact clustering, whereas the other states show a wider distribution indicative of increased structural heterogeneity (Figure 3a).

**Figure 3:**
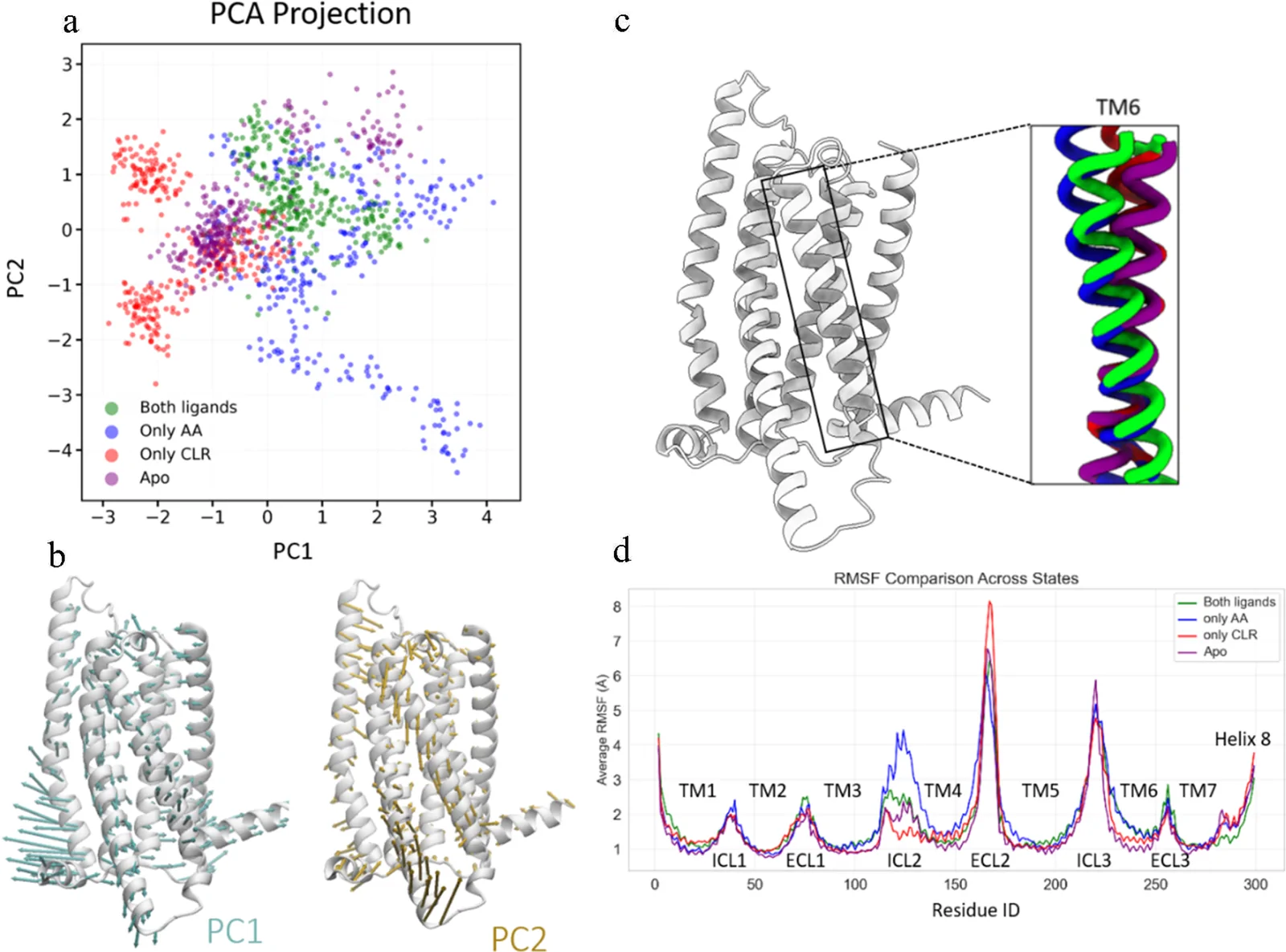
Principal component analysis (PCA) and RMSF analysis of TAS2R14 conformational dynamics. (a) PCA projections of TAS2R14 in different binding states, showing the overall conformational space and individual state distributions. (b) Conformational change along PC1/PC2. (c) Structural comparison highlighting the major conformational variations between states, primarily involving movements of transmembrane helix 6 (TM6). Representative structures were extracted from distinct PCA clusters at the following simulation time points: Apo (460 ns), both ligands (660 ns), only CLR (1999 ns), and only AA (1220 ns). (d) RMSF of the receptor for all states. Transmembrane (TM) and loop regions are annotated.

RMSF analysis revealed small, localized differences, particularly in Intracellular loop 2 (ICL2), where CLR showed a stabilizing effect and in transmembrane helix 6 (TM6), where AA showed a destabilizing effect (Figure 3d).

ECL2 is the longest and least conserved extracellular loop among Class A GPCRs and has been shown to play important roles in ligand selectivity and receptor activation, displaying distinct conformational behaviors across different receptors.^43^ ECL2 was unresolved in the experimental cryo-EM structure and reconstructed computationally using TAS2R14 Alphafold^24^ model. In our simulations, ECL2 exhibits elevated flexibility (Figure 3d), a feature typical of intrinsically disordered loop regions in GPCRs.^44^ Notably, this flexibility varies between binding states, highlighting its possible role in accommodating different ligands.

PCA shows that in AA bound states, TM6 moves outward in the bottom region and inward at the top, unlike the states with no AA (Figure 3c). To put this specific movement into the context of general receptor activation, it is worth comparing it to other GPCR families. TM6 movement has an important role in GPCR activation.^45^ In the inactive conformation of Class A GPCRs, the highly conserved R (3.50) residue of the DRY motif typically forms a salt bridge, known as the ionic lock, with a negatively charged residue in TM6, usually E or D (6.30). This interaction holds TM6 closer to TM3, thereby stabilizing the receptor in its inactive state. Upon agonist binding, conformational rearrangements disrupt this TM3-TM6 interaction, breaking the ionic lock and allowing TM6 to swing outward in the intracellular side. This outward displacement opens the receptor’s cytoplasmic interface, enabling G protein binding and activation. In most GPCR classes, this is driven by a pronounced movement of TM6. For example, in Class A GPCRs, a conserved proline (6.50) induces a characteristic kink in TM6, which straightens and swings outward during activation, creating space for G protein binding.^46^ In contrast, TAS2R14 exhibits a different behavior. As TAS2Rs lack this conserved proline,^47^ the TAS2R14 TM6 does not undergo the typical sharp kink observed in Class A GPCRs. Instead, the opening of the intracellular cavity involves a more subtle and continuous displacement of TM6, resembling the smoother structural rearrangement described for Class F GPCRs.^46^ This smoother, proline-independent TM6 displacement may facilitate intracellular conformational rearrangements and may potentially contribute to the structural flexibility associated with the broad ligand recognition profile of TAS2R14. This continuous outward movement of TM6 is accompanied by the expansion of the intracellular binding cavity, as evidenced by the strong positive correlation between TM3-TM6 distance and pocket volume across all states (Figure S6).

### TM6 Motion and Water Behavior

To characterize ligand-dependent changes in the intracellular cavity, we measured the distance between Y107 (3.50), a residue of the conserved DRY motif observed in Class A GPCRs,^47^ and V230 (6.35) based on the structural equivalents of the ionic lock motif discussed above (defined via structural alignment with the β2-adrenergic receptor^48^). This distance is longer in the AA-bound structures, while states lacking AA display a shorter distance (Figure 4a). Interestingly, in the complex containing only CLR in the extracellular site, transient peaks in this distance were observed after ∼1 μs. These fluctuations correlate with increased TM3-TM6 flexibility in the presence of CLR, which is consistent with previous experimental observations that cholesterol can weakly activate TAS2R14 signaling.^9^ We next analyzed the presence of water in both the extracellular (Figure S7) and intracellular sites (Figure 4c,d). In accord with the volume and TM3-TM6 distance, complexes containing AA showed higher water occupancy in the intracellular site, with the dual-binder complex exhibiting the greatest water content. The increased water occupancy colocalized with AA presence suggests that the expanded intracellular cavity becomes hydrated upon agonist engagement, possibly facilitating ligand entry or stabilizing the bound pose.

**Figure 4:**
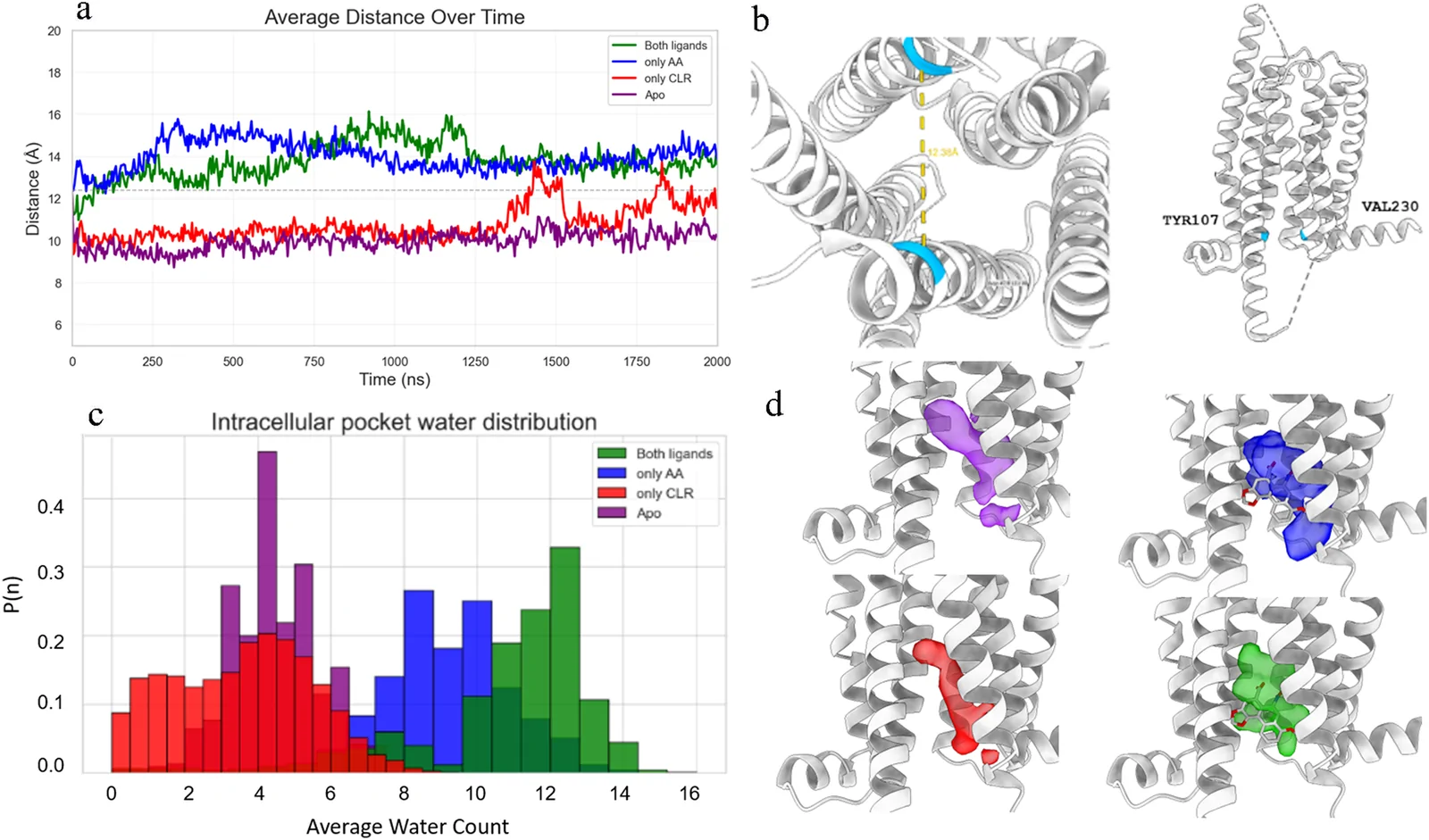
Distance between VAL230 and TYR107 in TAS2R14. (a) Distance between VAL230 (6.35) and TYR107 (3.50) Cα atoms in all states as a function of simulation time. The dashed line at 12.38 Å marks the distance in the original solved structure. (b) Location of the residues TYR107 (3.50) and VAL230 (6.35) and the distance in the original solved structure (8XQO), also highlighted in (a). (c) Probability distributions of the number of water molecules within 3 Å of the ligand or equivalent empty pocket volume in the intracellular site. (d) Representative water occupancy maps generated with VolMap.^38^

### Chloride Ion Binding to TAS2R14

In Class A GPCRs, a conserved sodium-binding pocket has been shown to stabilize the inactive receptor state by coordinating sodium entering from the extracellular site, with the negatively charged D(2.50), polar N(7.49), and neighboring residues.^49^ In TAS2R14, the same positions are occupied by positively charged residues, R55 (2.50), shown by MM/GBSA calculation to be important in AA binding (Figure S3), and H276 (7.49), which are highly conserved (∼96%) across hTAS2Rs (Figure S8). We analyzed the behavior of sodium and chloride ions in our system (50 mM NaCl). Since our simulations use a symmetric ionic environment, these findings represent structurally accessible pathways rather than physiological ion flux. Under our simulation conditions, which employed a fixed-protonation state for all residues, sodium ions did not exhibit particular interactions with the receptor in any of the simulated states, but a chloride ion entered from the intracellular side and migrated upward to interact with R55 and H276 in the absence of AA but in the presence of CLR (Figures 5a, S9). This represents an inverted charge configuration compared to Class A GPCRs, where positively charged sodium can bind to the negatively charged D(2.50), while in TAS2R14, negatively charged chloride interacts with the positively charged R(2.50). Within the constraints of this fixed-protonation framework, electrostatic surface analysis revealed that the intracellular surface of TAS2R14 is predominantly positively charged, creating a favorable electrostatic pathway that may facilitate chloride entry from the cytoplasmic side and guide the ion toward the positively charged residues R55 (2.50) and H276 (7.49) (Figure 5c and supporting movie).

**Figure 5:**
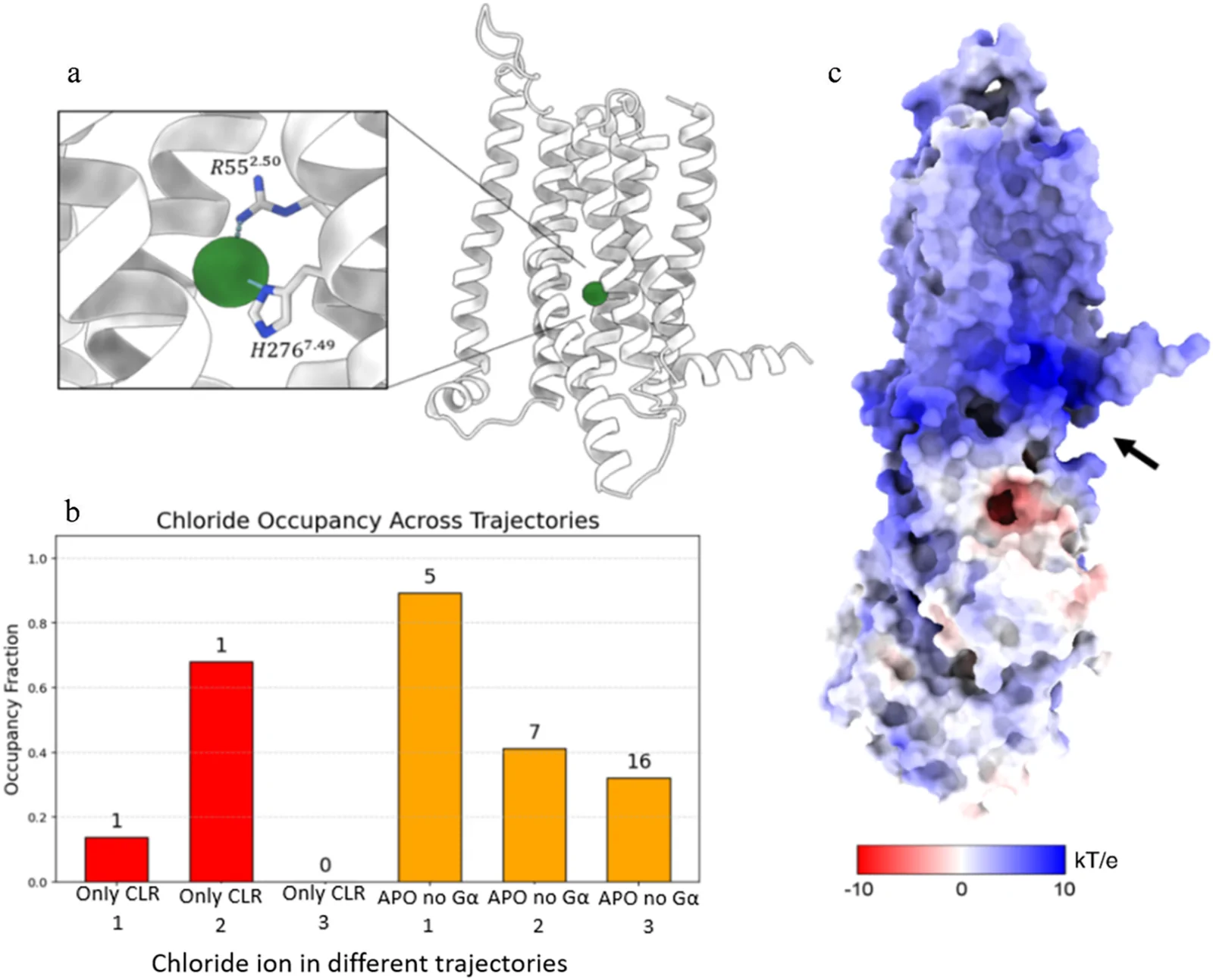
Chloride ion binding within TAS2R14. (a) Location of the chloride ion and its interactions with R55 (2.50) and H276 (7.49). (b) Occupancy of the potential chloride pocket throughout the simulations in which it was observed: only CLR (red) and Apo without Gα (orange). The number above each bar indicates the number of different chloride ions that occupied the pocket in each simulation. (c) The receptor surface is colored according to electrostatic potential calculated using APBS, with positive potential shown in blue and negative potential in red (−10 to +10 kT/e) showing positive charge at the area where chloride enters (shown by arrow).

Chloride binding occurred exclusively in the only CLR simulations and was absent in both the Apo (with Gα) and AA-bound systems. This pattern suggests that (i) the intracellular cavity must remain sufficiently open to permit ion entry, and (ii) cholesterol facilitates such accessibility, as supported by the increasing Y107–V230 distance observed during the CLR-bound trajectories (Figures 4 andS6).

To assess whether Gα occludes the intracellular entry pathway for chloride, we performed additional Apo simulations with the G protein subunit removed (Apo no Gα). In three independent replicates we again observed chloride entry into the same region. In the Apo simulations without Gα, the intracellular pocket remained open, and chloride ions were repeatedly observed entering and leaving the cavity; however, they did not form stable interactions. Instead, different chloride ions transiently occupied the site, with variable residence times and overall occupancies across trajectories (as indicated by the number of entering chloride ions shown above each bar; see Table S4 for detailed ion binding). In contrast, in the only CLR state, a single chloride ion entered and remained stably bound throughout two out of three trajectories (Figure 5b, Table S4, Figure S9).

Taken together, these findings suggest that chloride can interact with residues inside the receptor, above the newly discovered intracellular pocket. The entrance becomes accessible in specific observed conditions and open conformations of TAS2R14, particularly those favored by cholesterol binding, or in the absence of Gα. Notably, based on the binding patterns we observed, the role of the chloride may be more prominent when the extracellular site is occupied by CLR but the intracellular site is free. The ion’s presence in both the Apo (without Gα) and only CLR states indicates that chloride is more likely to enter without intracellular ligand presence. When the entry is not blocked by a ligand, the negative ion is engaging the positively charged residues R55 and H276. A recent study experimentally explored the effects of sodium chloride on intracellularly bound ligands (AA and flufenamic acid) and showed almost no change in activation when exposed to increasing externally supplied concentrations of sodium chloride. The activation of another bitter taste receptor, TAS2R16, by its extracellularly bound agonist salicin, was slightly reduced by sodium chloride supplementation.^50^ These observations motivate future experimental and computational studies of ligand-dependent modulation of ion accessibility and of potential functional roles of intracellular chloride on TAS2R signaling. Because the electrostatic properties of this region, especially around H276, may depend on local pH and protonation states, our current simulations should be interpreted within the limits of the predefined charge distribution used here. In the human body, chloride has a higher concentration in the extracellular matrix than in the cytosol, which makes the observed entrance of the ion from the intracellular site even more interesting. However, intracellular chloride levels are highly dynamic and tissue-specific, typically ranging from 5 to 40 mM in mammalian cells^25^ and reaching higher levels (∼50 mM) in specific sensory systems.^26,27^ Moreover, the chloride concentration is different in different organelles inside the cell, and the control of concentrations is mostly done by ion channels.^51^

Simulations at varying chloride concentrations, longer time scales, and different histidine protonations^52^ and constant-pH MD^53^ will be needed to further explore this phenomenon. With the current surge in cryo-EM structures of bitter taste receptors and their ligands,^16,17^ including TAS2R14 complexed with tangeretin in the canonical extracellular site (PDB: 9W0Q),^55^ experimental and computational exploration of the roles of ions across conditions, ligands and members of the TAS2R family is now becoming possible.

## Conclusions

2 μs long MD simulations revealed the flexibility of TAS2R14 binding sites, showing that both expand upon ligand binding. This property may underlie the receptor’s ability to accommodate a highly diverse set of ligands.^4^

Our results suggest that the extracellular ligand influences the intracellular site, as shown by the conformational coupling between binding pockets and the TM3-TM6 distance. Since cholesterol has been suggested to act as a weak agonist,^9^ it is reasonable to propose that its binding induces partial opening of the intracellular region without enabling full receptor activation. This is further evidenced by our PCA and RMSF analyses, which reveal conformational differences between binding states within TM6. Notably, TM6 adopts opening patterns distinct from those seen in most Class A GPCRs, particularly in the presence of the intracellular agonist aristolochic acid. The AA-bound simulations consistently show increased TYR107 (3.50)-VAL230 (6.35) distances, indicating that ligand occupation at the intracellular site locally promotes an expanded conformation of the receptor’s cytoplasmic region.^44^

Further insight came from analysis of chloride ion behavior. Chloride binding occurred in the only CLR and the Apo without Gα states, and was absent in both the Apo with Gα and AA-bound systems. Combined with the observed increase in the TYR107 (3.50)-VAL230 (6.35) distance and increased pocket volume, this suggests that chloride entry occurred when the intracellular cavity remained more accessible, particularly in the CLR-only state. This is interesting in view of the observations reported for Class A GPCRs, where sodium helps stabilize the inactive state.^49^

Our findings indicate ligand-dependent covariation of pocket volume and intracellular structural descriptors between TAS2R14 binding sites, and a charge-inverted (compared to Class A) positively charged intracellular region around residue 2.50 that was accessible to chloride under the simulated conditions. The observed dynamics obtained under the specific simulation conditions open new questions on the role of negative ions in the intracellular environment and their potential influence on receptor function and physiological role. In the meantime, these observations highlight the TAS2R-specific arginine in position 2.50, and the intracellular region of TAS2R14 as a potential site for designing modulators of this pocket for TAS2R14 regulation.

## Supplementary Material









## Data Availability

All files required to reproduce the molecular dynamics simulations are publicly available via Zenodo. The deposited data sets include starting protein and ligand structures (PDB), GROMACS coordinate and topology files generated using CHARMM-GUI (GRO, TOP, ITP), and simulation parameter files for minimization, equilibration, and production runs (MDP). Simulation files are available for the following systems: simulations with both ligands (10.5281/zenodo.18287809), only cholesterol (10.5281/zenodo.18288174), only aristolochic acid (10.5281/zenodo.18288127), apo receptor with Gα (10.5281/zenodo.18288089), and apo receptor without Gα (10.5281/zenodo.18288040). The molecular dynamics simulation trajectories generated in this study have been deposited in Zenodo: APO with Gα link: 10.5281/zenodo.18150976. Only CLR private link: 10.5281/zenodo.18105160.

## Notes

Time after initiation of the trajectory: 1600 ns (MP4)
supporting video 2 Protein conformational motion along PC1 (MP4)
supporting video 3 Protein conformational motion along PC2 (MP4)
Molecular dynamics minimization protocol (Table S1); molecular dynamics equilibration protocol (Table S2); Description of simulated receptor states and trajectories (Table S3); validation of ligand force-field parameters by comparison of MD-derived and reference ligand structures (Figure S1); RMSD analyses of receptor and ligand stability across simulation states (Figure S2); residue contact frequencies and MM/GBSA binding energy decomposition for cholesterol and aristolochic acid (Figure S3); RMSF profiles of TAS2R14 across simulated states (Figure S4); TM3–TM6 distance trajectories for individual simulation replicates (Figure S5); correlation between intracellular pocket volume and TM3–TM6 distance (Figure S6); water occupancy analysis of the extracellular binding pocket (Figure S7); conservation of residues at Ballesteros–Weinstein positions 2.50 and 7.49 across human TAS2Rs (Figure S8); chloride ion occupancy, entry events, and residence times in the intracellular pocket (Table S4); chloride ion binding kinetics and representative ion trajectories (Figure S9) (PDF)
