# Supplementary material for: Dynamics of Ligand Binding Sites and Chloride Penetration in a Bitter Taste GPCR
Source: J Chem Inf Model. 2026 Sep 3;66(18):11981–90. doi: 10.1021/acs.jcim.6c00140 (PMC13625955; doi:10.1021/acs.jcim.6c00140)
Supplement: Supplementary file 1 [file ci6c00140_si_013.pdf]

Supplementary information for:

# Dynamics of Ligand Binding Sites and Chloride Penetration in a Bitter Taste GPCR

Alon Rainish[1], Liel Sapir[2*] and Masha Y Niv[1*]

[1]The Institute of Biochemistry, Food Science and Nutrition, The Robert H. Smith Faculty of Agriculture, Food and Environment, and The Fritz Haber Center for Molecular Dynamics, The Hebrew University, Israel. [2]Department of Chemistry and the Institute of Nanotechnology and Advanced Materials, Bar-Ilan University, Israel.

**Protonation states of histidine residues assigned by MolProbity:**
HSD refers to histidine with a proton on the delta nitrogen; HSE refers to histidine with a proton on the epsilon nitrogen. All assignments were optimized to minimize steric clashes and maximize hydrogen-bonding networks.

Receptor (chain R): HSE94, HSD151, HSE208, HSE214, HSE227, HSD276
Gα (chain A): HSE188, HSE195, HSE213, HSE244, HSE322

Simulation steps

Table S1- minimization steps

| step | nsteps | Integrator | emtol (kJ·mol⁻¹) | Pos. restraints BB / SC / Lipid (kJ·mol⁻¹·nm⁻²) | DIHRES | DIHRES_FC (kJ·mol⁻¹·nm⁻²) | Constraint |
|---|---|---|---|---|---|---|---|
| 1 | 5000 | steep | 1000.0 | 4000 / 2000 / 1000 | Yes | 1000.0 | h-bonds (LINCS) |
| 2 | 50000 | steep | 0.001 | 2000 / 1000 / 500 | Yes | 500.0 | h-bonds (LINCS) |
| 3 | 50000 | steep | 0.001 | 250 / 500 / 1000 | Yes | 250.0 | h-bonds (LINCS) |
| 4 | 50000 | steep | 0.001 | 0 / 250 / 500 | Yes | 0.0 | h-bonds (LINCS) |
| 5 | 50000 | steep | 0.001 | 0 / 0 / 0 | Yes | 0.0 | h-bonds (LINCS) |

-LINCS was used for all hydrogen bond constraints throughout the simulation.
-Pos. restraints: positional restraints applied to backbone (BB), sidechains (SC), and lipids.
-DIHRES: dihedral restraints applied (yes/no).
-DIHRES FC: force constant for dihedral restraints.
-All abbreviations are defined in the column headers; energy units are kJ·mol⁻¹ and distances in nm⁻².

Table S2- equilibration steps

| step | BB restr. (KJ/mol*nm2) | SC rerst. (KJ/mol*nm2) | Lipid restr. (kJ/mol*nm2) | Thermostat | Barostat | Timestep (fs) | Duration (ns) |
|---|---|---|---|---|---|---|---|
| 1 | 4000 | 2000 | 1000 | v-rescale | / | 1 | 0.125 |
| 2 | 2000 | 1000 | 400 | v-rescale | / | 1 | 0.125 |
| 3 | 1000 | 500 | 400 | v-rescale | C-rescale | 1 | 0.125 |
| 4 | 500 | 200 | 200 | v-rescale | C-rescale | 2 | 0.5 |
| 5 | 200 | 50 | 40 | v-rescale | C-rescale | 2 | 0.5 |
| 6 | 50 | 0 | 0 | v-rescale | C-rescale | 2 | 0.5 |

Table S3- trajectories description

| Trajectory name | Descriptions | Gα subunit | Ligand |
|---|---|---|---|
| Both ligands | CLR at the extracellular site and AA at the intracellular site<br>Color code: green | Yes | cholesterol and Aristolochic acid |
| Only CLR | CLR at the extracellular site<br>Color code: red | Yes | Cholesterol |
| Only AA | AA at the intracellular site<br>Color code: blue | Yes | Aristolochic acid |
| Apo with Gα | Apo with Gα<br>Color code: purple | Yes | - |
| Apo no Gα | Apo without Gα<br>Color code: orange | No | - |

**Ligand integrity**

A representative structure of the ligand was extracted from the equilibrated MD trajectory using the gmx trjconv utility. Structural alignment between this MD-derived geometry and the initial reference coordinates from the solved structure PDB yielded an RMSD of 0.386 Å for the core of AA and 0.659 for CLR (Figure S1). This low deviation confirms the high structural fidelity of the force field and the accuracy of the bonded parameters, consistent with the stable RMSD profiles observed across the production runs (Figure S2).

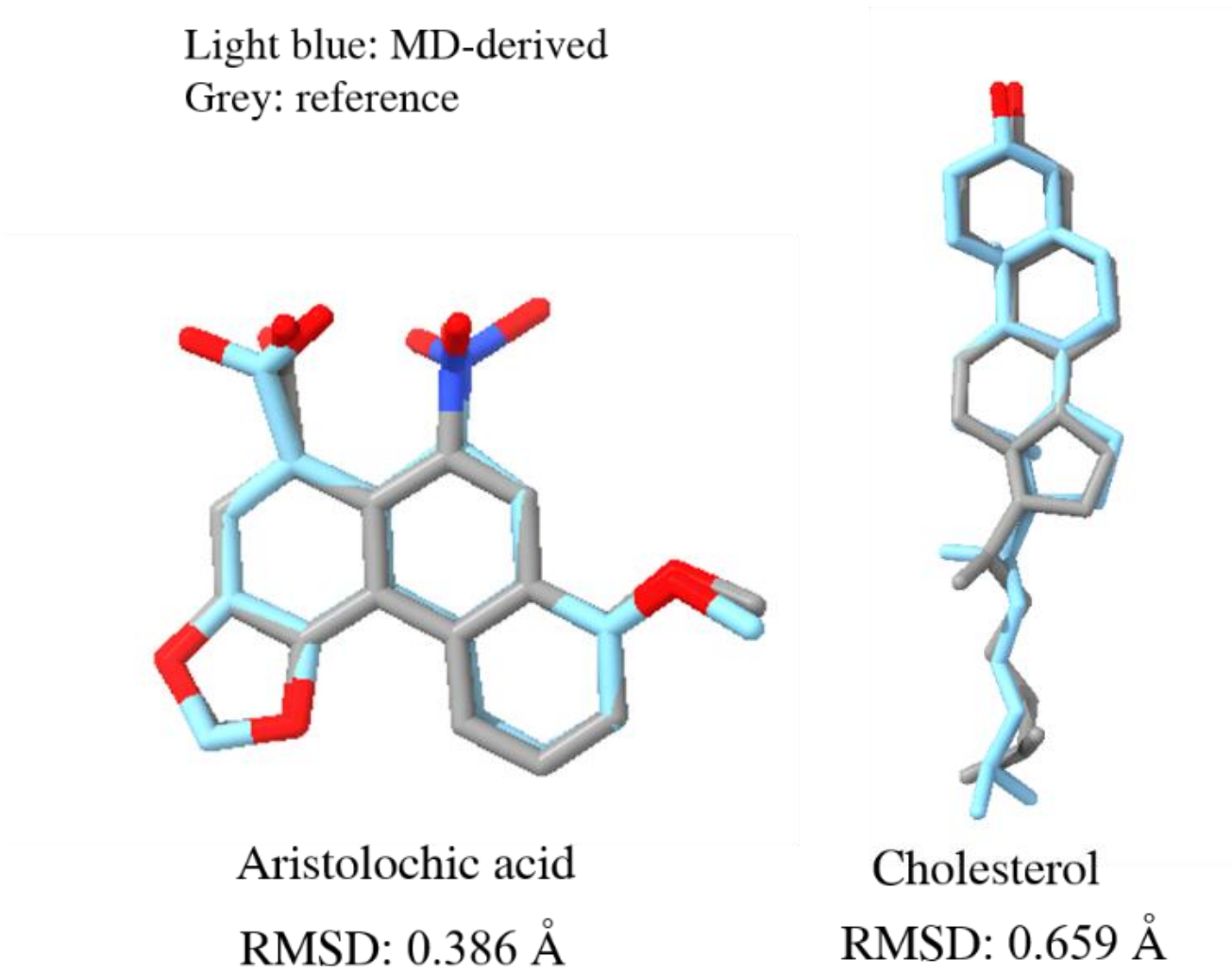


**Figure S1. Structural validation of ligand parameters**. Overlay of the MD-derived structures (light blue) and the initial reference structures (grey) for ligands AA (left) and CLR (right). The low RMSD values (0.386 Å and 0.659 Å, respectively) confirm that the CGenFF force field parameters generated via CHARMM-GUI accurately maintain the structural integrity and conformational preference of both ligands throughout the simulation.

**Povme coordinates**

For the extracellular site: A spherical inclusion region with a radius of 4 Å was defined around the center of the pocket to enclose the binding site. The grid spacing was set to 1.0 Å, and a distance cutoff of 1.09 Å was used to determine inclusion of grid points within the pocket.

For the intracellular site: A spherical inclusion region with a radius of 6 Å was defined around the center of the pocket to enclose the binding site. The grid spacing was set to 1.0 Å, and a distance cutoff of 1.09 Å was used to determine inclusion of grid points within the pocket.

Volumes were computed for every 200 frames of the simulation trajectory, and the resulting data were averaged across frames to assess pocket dynamics over time.

**RMSD**

Ligand RMSD analysis revealed distinct mobility patterns depending on the binding state (Fig. S2). Ligand RMSD, calculated with the same alignment, primarily captures translation and rotation within the binding site rather than conformational changes of the ligands themselves. In the intracellular site, AA exhibited lower RMSD when bound alone, indicating greater stability in the absence of CLR. When CLR was present in the extracellular site, AA's RMSD increased, suggesting that ligand occupancy in the opposite site enhances AA's mobility rather than stabilizing it (Figure S2.f). Towards the end of the trajectory of one replica of the only AA trajectories, AA was able to leave the site (Figure S2.b), thereby explaining the hump in its RMSD. A similar trend was observed in the extracellular site, where CLR was more stable when bound alone, while its RMSD increased when AA was simultaneously bound intracellularly (Figure S2.e).

Cα RMSD analysis indicated comparable overall stability of the receptor across all states, with the dual-ligand state showing slightly higher stability over time (Figure S2.g). Structures were aligned to their reference conformation, so receptor RMSD reflects internal conformational changes. These results indicate that ligand-binding status affects ligand positioning within the pockets but has minimal impact on overall receptor stability.

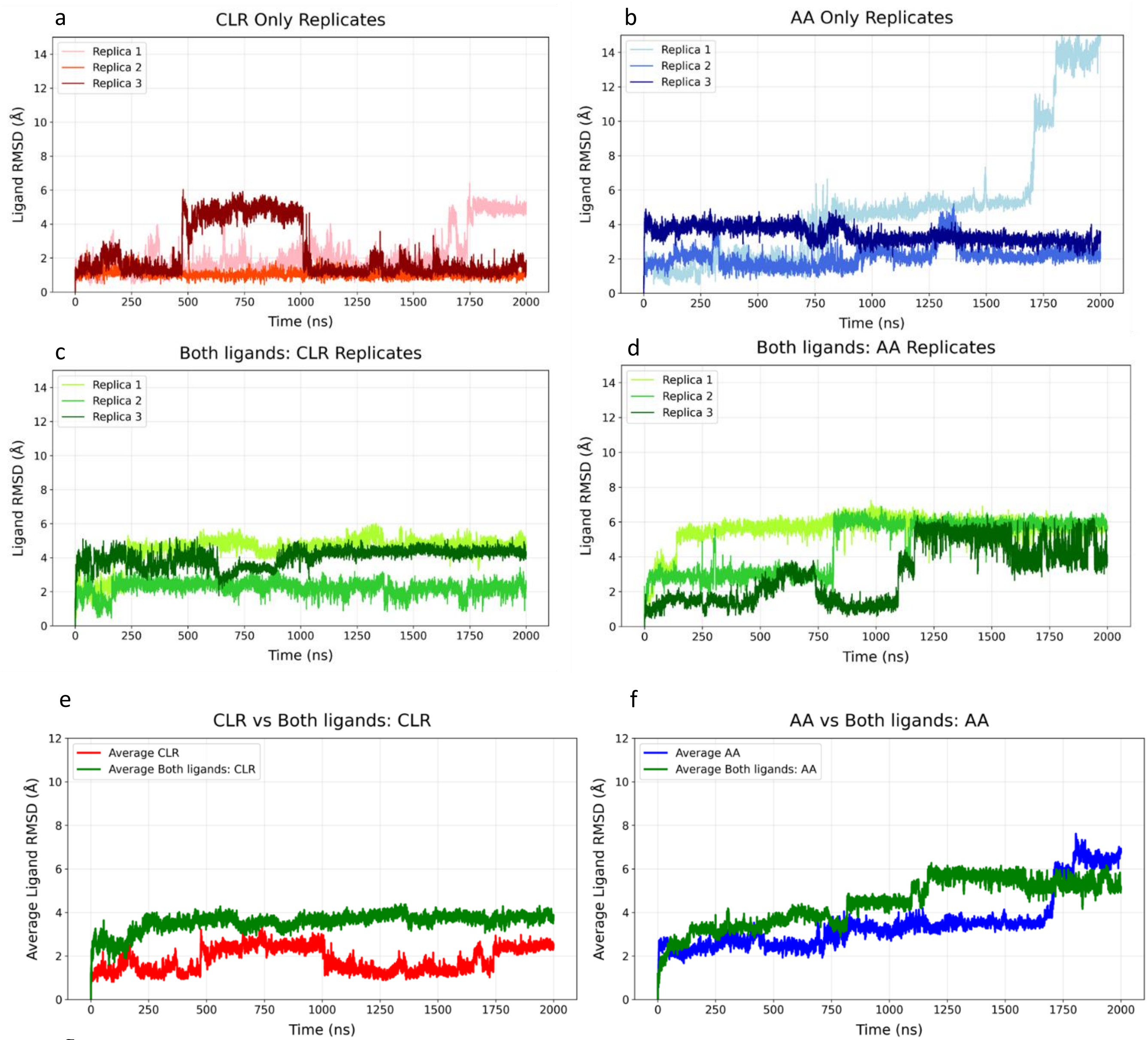


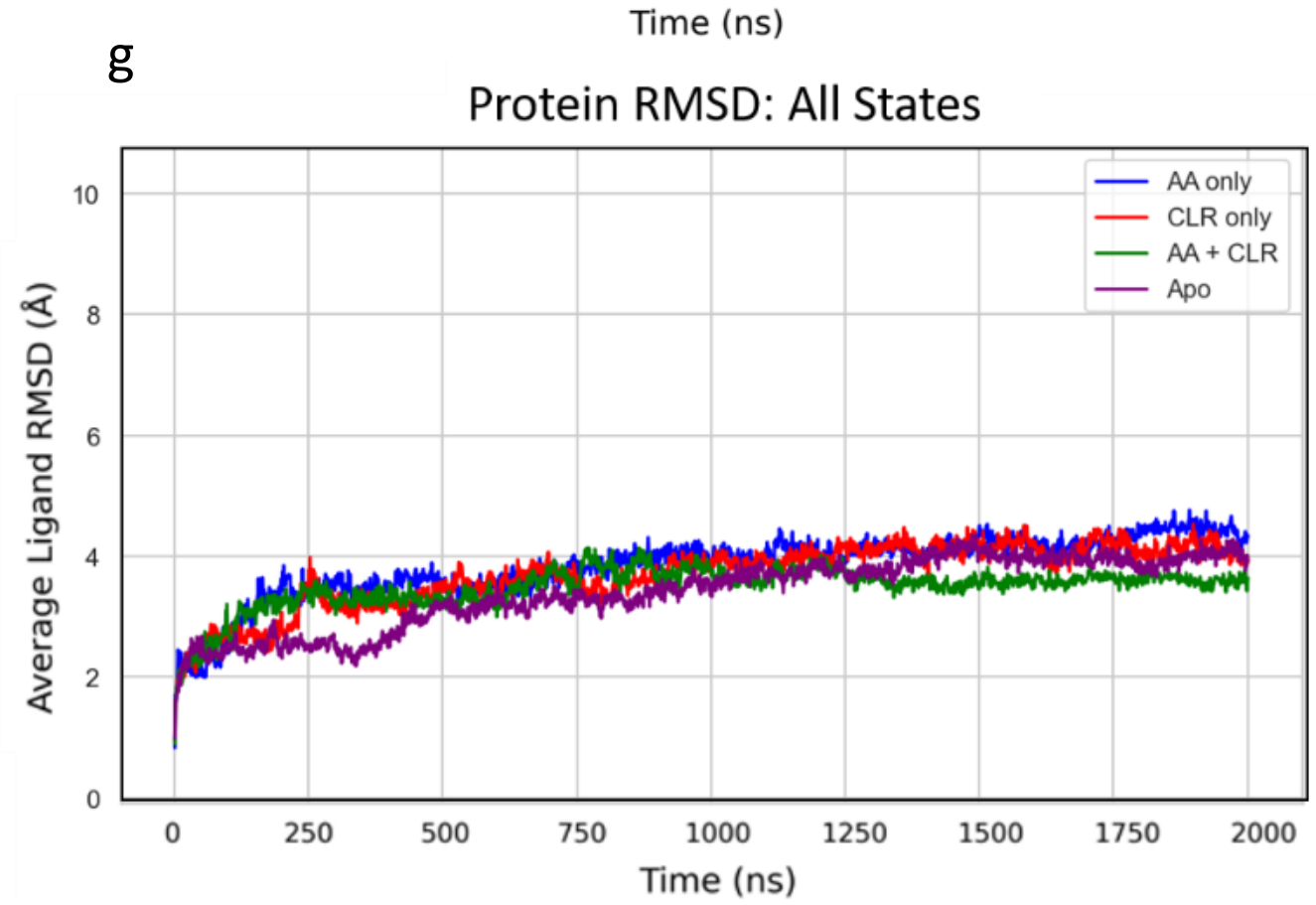


**Figure S2. RMSD analysis of TAS2R14 receptor and ligands.** (a) RMSD of three replicates of CLR in only CLR state. (b) RMSD of three replicates of AA in only AA state. (c) RMSD of three replicates of CLR in Both ligands state. (d) RMSD of three replicates of AA in Both ligands state. (e) RMSD average of CLR when bound alone (red) or together with AA in the intracellular site (green). (f) RMSD average of AA when bound alone (blue) or together with CLR in the extracellular site (green). (g) Cα RMSD of the receptor in all simulated states: only AA (blue), only CLR (red), Both ligands (green), and Apo with Gα (purple).

**MM/GBSA binding energy and Ligand contact**

To address the stability and energetic basis of the ligand-receptor interactions, we analyzed residue-specific contact frequencies alongside MM/GBSA binding energy decomposition. The results demonstrate that CLR remains firmly anchored in the binding pocket throughout the simulations, maintaining near-constant contact (frequency ~100%) and strong energetic stabilization with a core set of residues, including TRP89 (3.32), VAL180 (5.40), and SER265 (7.38), regardless of the presence of the ligand (Figure S3, top). For AA, while the isolated system shows more transient interactions as directly evidenced by the lower contact frequencies and large error bars observed for residues such as THR101 (3.44) and MET197 (5.57) in the "Only AA" state (Figure S3, bottom), the addition of CLR significantly stabilizes its position within the pocket. This is evidenced by a simultaneous increase in contact frequency and favorable binding energy contributions from specific residues such as VAL97 (3.40), ALA100 (3.43), and THR101 (3.44), which reach high occupancy and energetic relevance only in the dual-ligand system. Furthermore, ARG55 (2.50) was identified as a primary energetic anchor for AA, maintaining high-strength interactions across states. These complementary structural and thermodynamic profiles confirm that both ligands remain sequestered within the designated binding site, with CLR further promoting the energetic stability of AA.

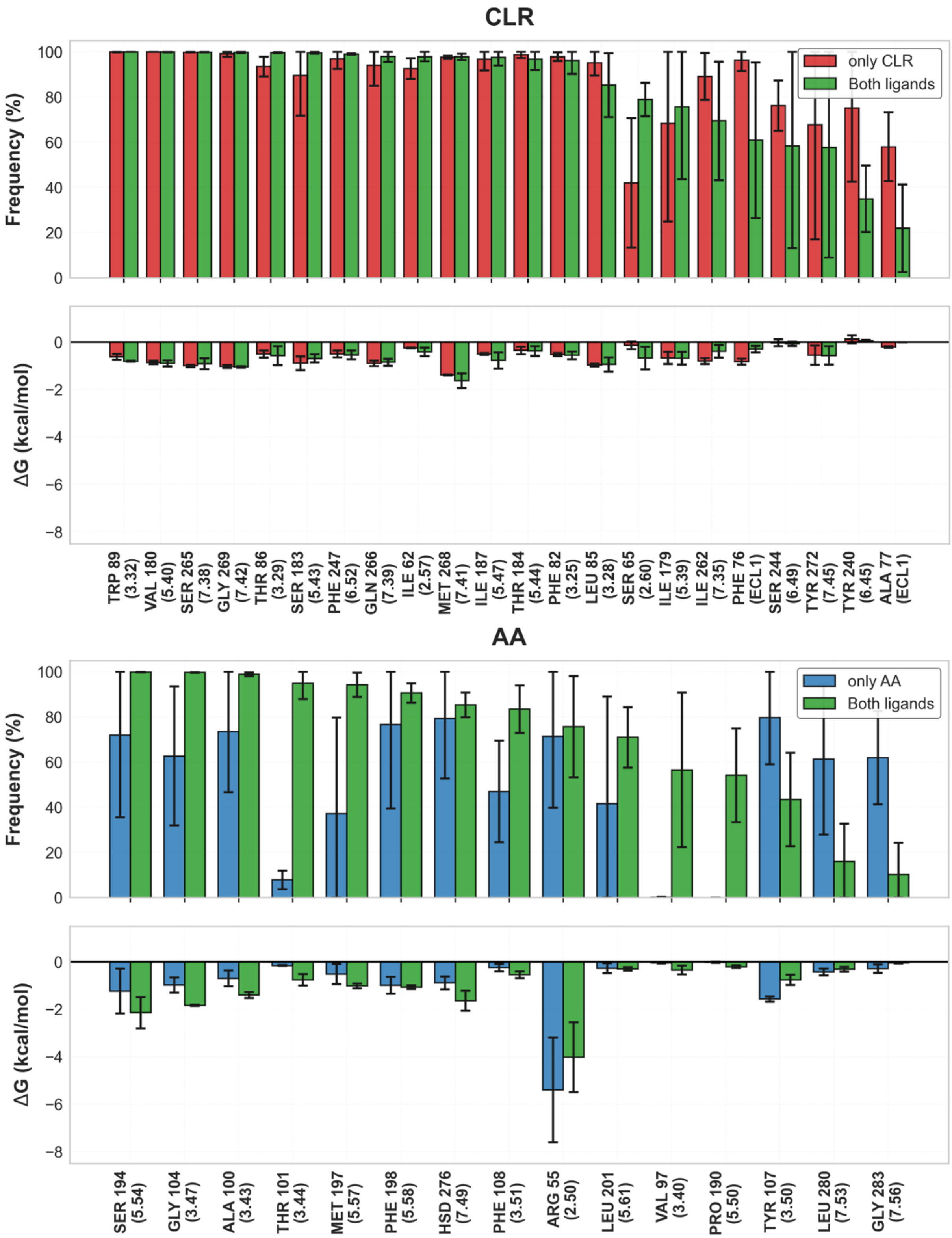


**Figure S3. Combined residue-specific contact frequency and binding energy decomposition for AA and CLR ligands.** Only residues with at least 50% occupancy/frequency in at least one of the tested states are shown. (Top) CLR binding site analysis: comparison of contact frequency (top panel, %) and binding energy decomposition (bottom panel, kcal/mol) between the isolated CLR system (red) and the dual-ligand system (green). (Bottom) AA binding site analysis: comparison of contact frequency (top panel) and MM/PBSA binding energy decomposition (bottom panel) between the isolated AA system (blue) and the dual-ligand system (green). Data represent the mean over triplicate MD trajectories, with error bars indicating the standard deviation. Residues are indexed by sequence number with their respective Ballesteros-Weinstein numbers provided in parentheses.

**RMSF**

Local receptor flexibility across all simulated conditions and replicates is summarized in the RMSF profiles shown in Figure S4. The differences between binding states were observed mostly in the following regions:

TM6: 227-252

ICL2: 112-125

ECL2: 162-172

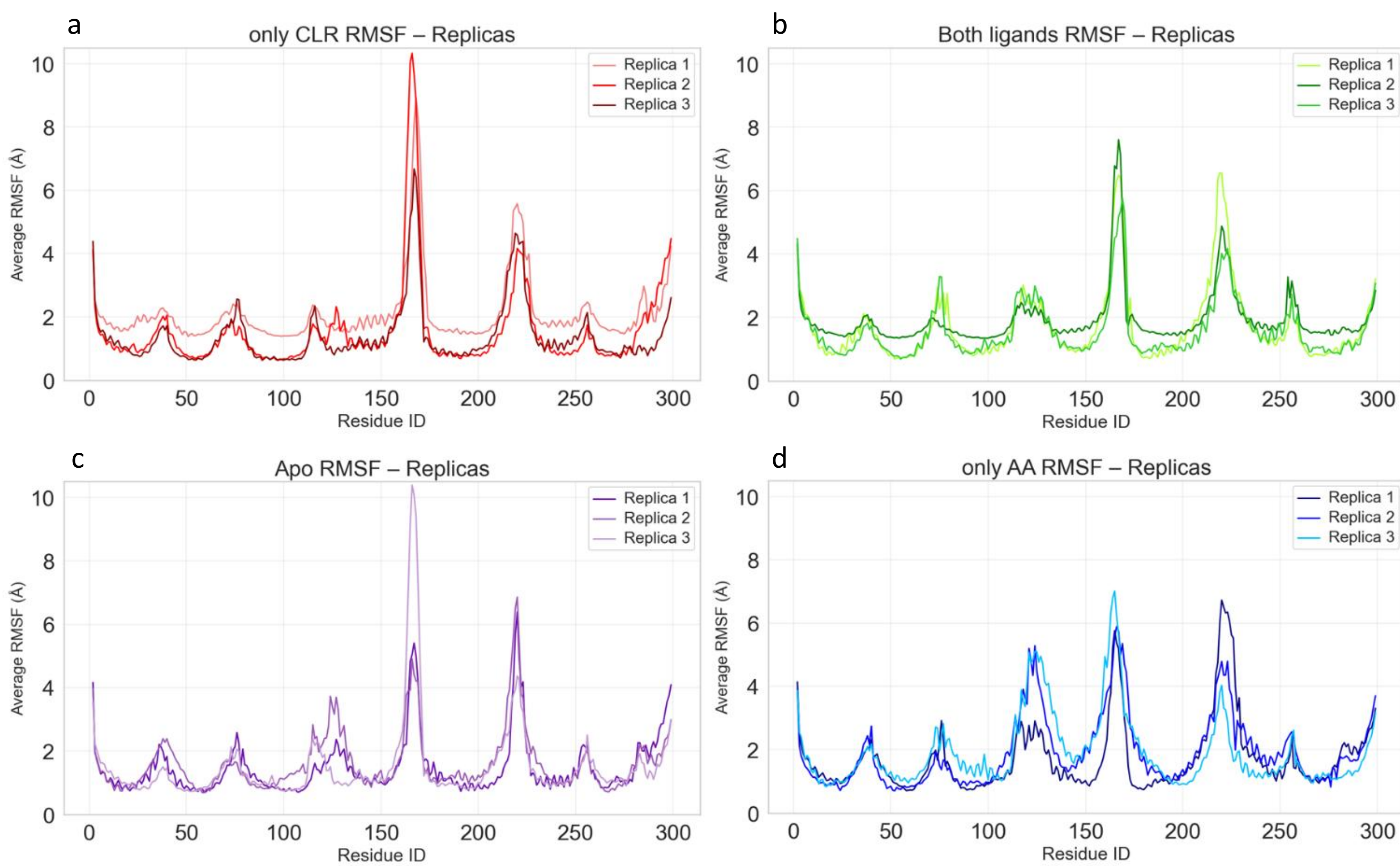


**Figure S4. RMSF of TAS2R14 across independent replicates.** RMSF profiles were calculated across three independent MD replicates to assess the local flexibility of the TAS2R14 receptor in four distinct states: (a) only CLR, (b) Both ligands bound, (c) Apo with Gα and (d) only AA. High fluctuation peaks correspond to the extracellular and intracellular loops (ECLs and ICLs), while troughs represent the stable transmembrane (TM) helices.

## Distance between VAL230 and TYR107

To ensure the robustness and reproducibility of our results, we examined the stability of the binding pocket distances across all independent replicates. While the main text presents the average values for clarity, the individual trajectories for each of the three 2,000 ns simulations are detailed here (Figure S5). These individual traces confirm that the observed distances are consistent across all runs. Importantly, these trajectories capture the structural dynamics of the TM3-TM6 distance, which is further analyzed for pocket-coupling correlations in Figure S6.

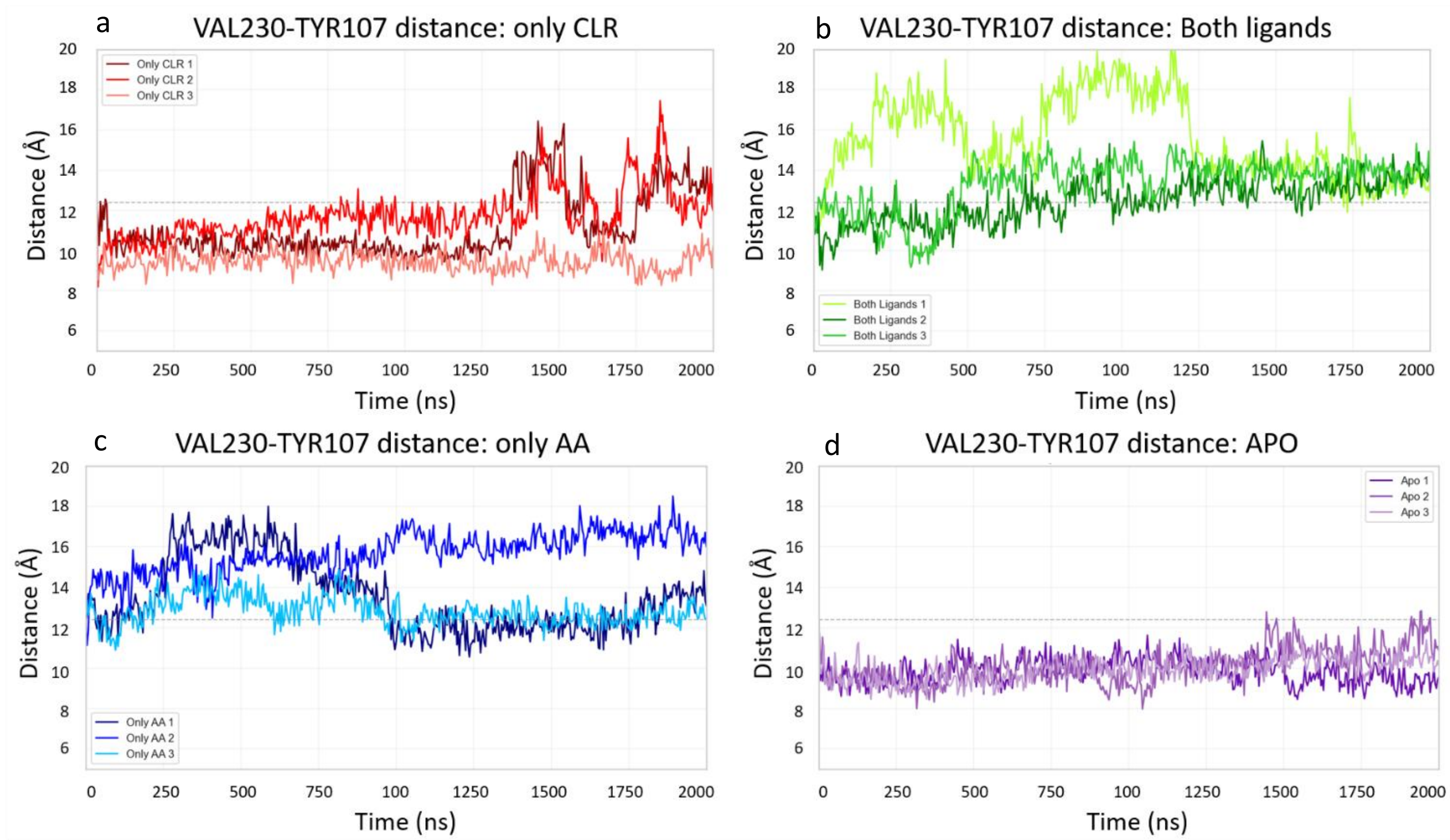


**Figure S5. TM3-TM6 distance across independent replicates.** Time-resolved distance (Å) between the alpha carbons of residues VAL230 and TYR107, monitored over 2,000 ns trajectories. Each panel displays the individual traces of three independent replicates for the following systems: (a) only CLR; (b) Both ligands; (c) only AA; and (d) Apo with Gα.

## Intracellular pocket volume and TM3-TM6 distance

To further investigate these structural transitions, we evaluated the correlation between the intracellular cavity volume and a physically interpretable structural metric, namely the TM3-TM6 distance (TYR107-VAL230 Cα distance). Our results reveal a strong, positive correlation across all simulated systems (Pearson R ranging from 0.60 to 0.86; Figure S6), indicating that variations in the intracellular pocket volume are structurally coupled to the relative displacement of TM6 (Figure S6).

To ensure these findings were not dominated by a single outlier trajectory, Pearson correlation coefficients were extracted for individual replicas. For the "Both ligands" system, R values were 0.475, 0.546, and 0.742 (replicas 1-3, respectively). For the "only CLR" system, robust positive correlations were consistently maintained across the successfully resolved trajectories (replica 1: 0.689; replica 2: 0.792). The "Apo no Gα" system yielded R values of 0.433, 0.743, and 0.432, while "only AA" system showed values of 0.793, 0.739, and 0.345. Every independent replica across all tested conditions consistently exhibited positive correlation trends, demonstrating that the structural co-variation is a reproducible feature of the simulation ensembles.

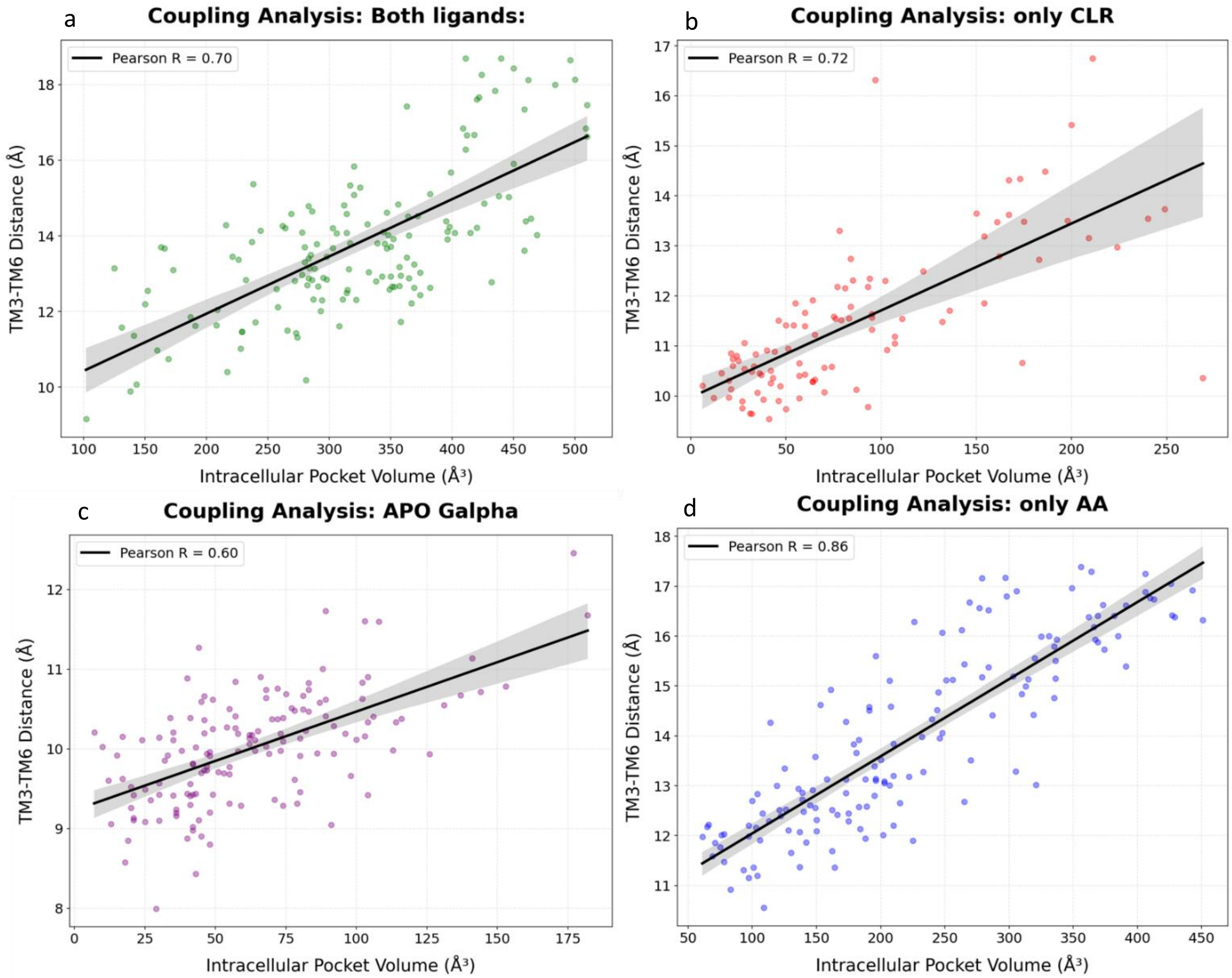


**Figure S6. Correlation between intracellular pocket volume and TM3-TM6 distance.** Scatter plots illustrating the correlation between the intracellular pocket volume (calculated via POVME3) and the distance between the intracellular ends of Transmembrane helices 3 and 6 (V230-Y107). Panels show: (a) both ligands, (b) only CLR, (c) APO state with Gα, and (d) only AA. Each plot includes the Pearson correlation coefficient (R), a linear regression line, and the confidence interval (shaded gray area).

## Water Occupancy

We were able to count a large number of molecules in the extracellular site in the states with no cholesterol present, while in the intracellular site, water molecules are in higher numbers in the states with AA in the pocket (Figure 4c). These opposing trends suggest that different hydration patterns govern each site's dynamics and ligand interactions. This pattern suggests that water molecules within the binding sites contribute to the binding profile of each site.

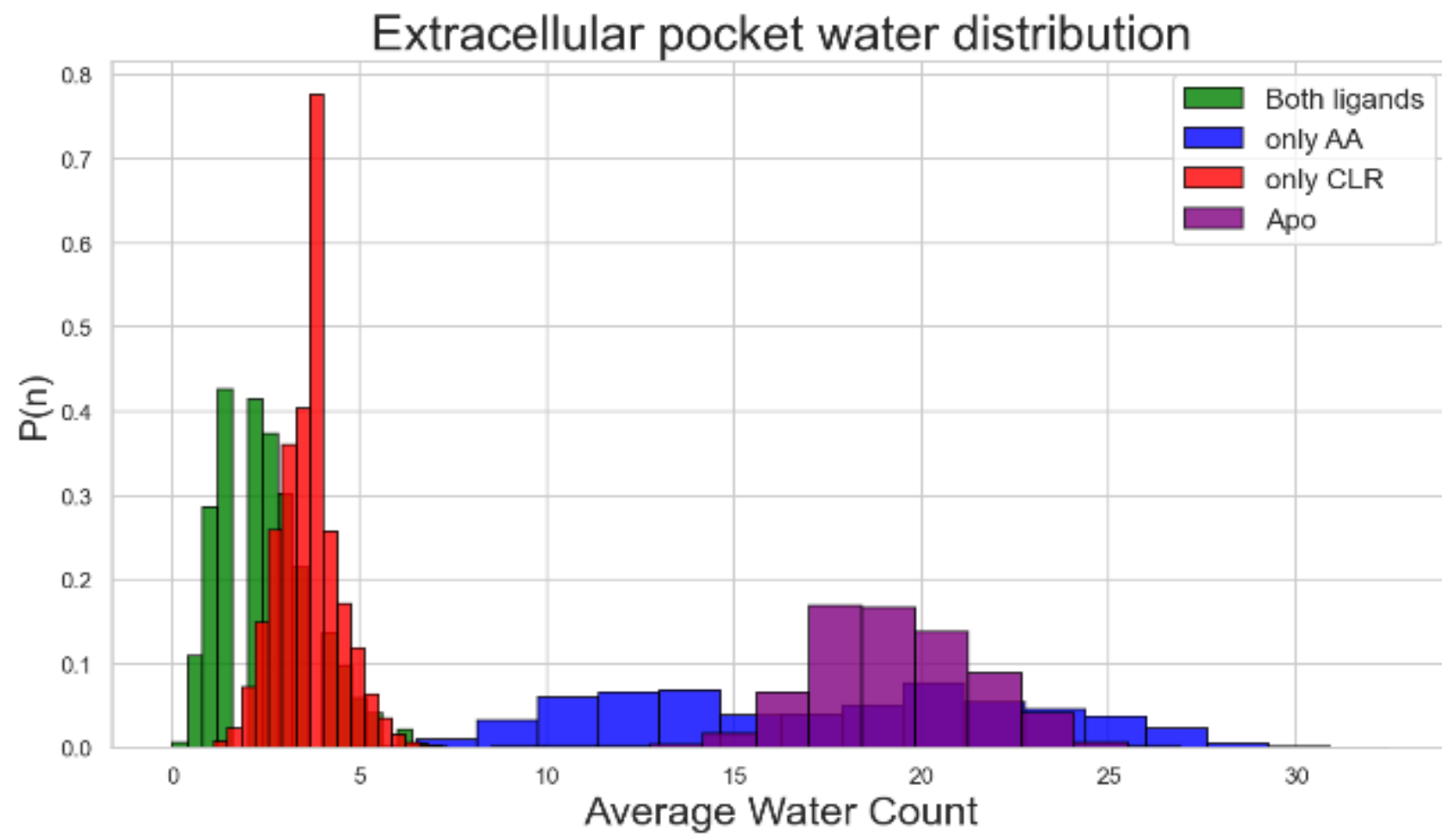


**Figure S7. Water molecule count in the extracellular site.** Number of water molecules within 3 Å of the ligand or equivalent empty pocket volume in the extracellular site. Colors correspond to the general color scheme.

| hTAS2R | 2.50 | 7.49 |
|---|---|---|
| 1 | R | H |
| 2 | R | H |
| 3 | R | H |
| 4 | R | H |
| 5 | R | H |
| 7 | R | H |
| 8 | R | H |
| 9 | R | H |
| 10 | R | H |
| 13 | R | H |
| **14** | **R** | **H** |
| 16 | R | H |
| 38 | R | H |
| 39 | R | H |
| 40 | R | H |
| 41 | R | H |
| 42 | T | H |
| 43 | R | H |
| 44 | R | H |
| 45 | R | H |
| 46 | R | H |
| 47 | R | H |
| 48 | R | H |
| 49 | R | H |
| 50 | R | D |
| 60 | R | H |
| **%** | **96.2%** | **96.2%** |

**Figure S8. Conservation of positions 2.50 and 7.49 across human TAS2Rs.** Multiple sequence alignment of human TAS2Rs showing residues at positions 2.50 and 7.49 (Ballesteros-Weinstein numbering). Each row represents a receptor, and the bottom row indicates percentage conservation.

Chloride ion binding events were defined using a 4.0 Å distance cutoff from residue R55 and a minimum residence time of 1.0 ns to exclude transient solvent collisions.

Table S4- Detailed chloride ion binding within the TAS2R14 pocket

| state | Number of Ions* | Entries | Max Residence Time (ns) | Avg Residence Time (ns) | Total Occ |
|---|---|---|---|---|---|
| Only CLR 1 | 1 | 38 | 62.2 | 7.0 | 0.142 |
| Only CLR 2 | 1 | 103 | 757.6 | 13.2 | 0.684 |
| Only CLR 3 | 0 | 0 | 0.0 | 0.0 | 0.001 |
| APO no Gα 1 | 5 | 47 | 200.4 | 38.3 | 0.904 |
| APO no Gα 2 | 6 | 197 | 24.2 | 4.0 | 0.472 |
| APO no Gα 3 | 12 | 59 | 239.4 | 10.7 | 0.334 |

*Ions count excludes transient collisions < 1.0ns

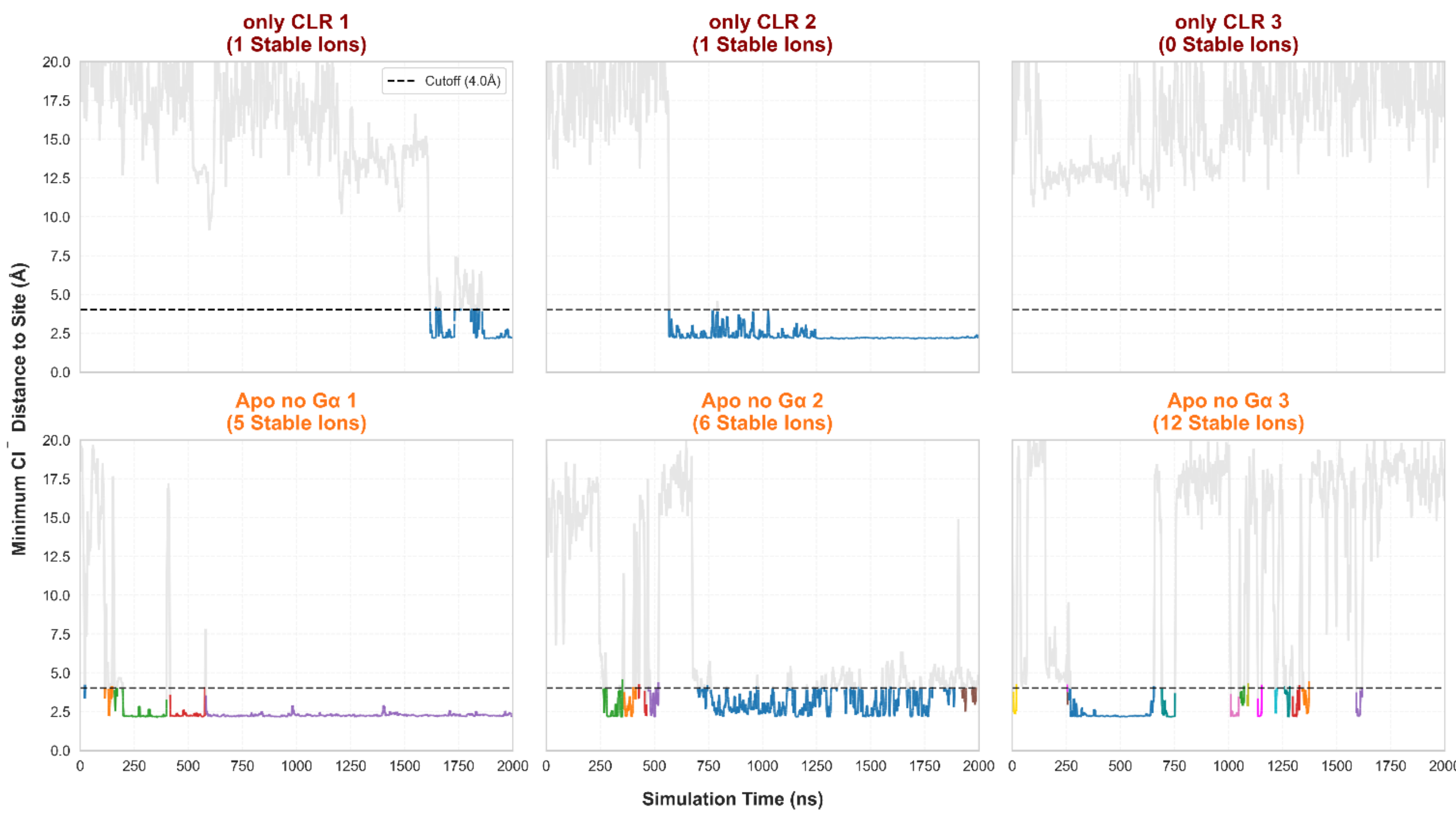


**Figure S9. Chloride ion binding kinetics and dynamic stability within the TAS2R14 pocket.** Minimum distance trajectories of Chloride ions to the binding pocket over 2000 ns, separated by state, only CLR (top) and Apo no Gα (bottom), across three independent replicates (columns). Lines under the 4.0 Å cutoff (dashed line) are dynamically colored by unique Ion IDs; a color change denotes ion replacement, while gray lines represent bulk solvent or transient collisions (<1.0 ns). Panel titles show the total stable ion count, matching Table S5 and highlighting the shift from high-turnover swapping (APO no Gα) to rigid single-ion trapping (only CLR).
